# Supplementary material for: Genomic Characterization of a Bataï Orthobunyavirus, Previously Classified as Ilesha Virus, from Field-Caught Mosquitoes in Senegal, Bandia 1969
Source: Viruses. 2024 Feb 6;16(2):261. doi: 10.3390/v16020261 (PMC10892164; doi:10.3390/v16020261)
Supplement: Supplementary file 1 [file viruses-16-00261-s001.zip › viruses-2699846-supplementary.pdf]

Table S1: Accession number of strains donwloaded on Genbank

**Strains**	**Accession S**	**Accession M**	**Accession L**	**Country**	**date of collection**
Batai virus isolate PV424	MH299972.1	MH299973.1	MH299974.1	Germany	2016
Batai virus strain Chittoor IG 20217	JX846598.1	JX846599.1	JX846600.1	India	1957
Batai virus strain CVOV 41.3	KM507321.1	KM507322.1	KM507323.1	Austria	2013
Batai virus strain Italy 2009	KC168046.1	KC168047.1	KC168048.1	Italy	2009
Batai virus strain MM2222	JX846595.1	JX846596.1	JX846597.1	Malaysia	1955
Batai virus strain NM 12	KJ187040.1	KJ187039.1	KJ187038.1	China	2012
Batai virus strain UgMP 6830	JX846601.1	JX846602.1	JX846603.1	Uganda	
Batai virus ZJ2014	KU746869.1	KU746870.1	KU746871.1	China	2014
Bunyamwera virus	NC_001927.1	NC_001926.1	NC_001925.1	NA	NA
Bunyamwera virus isolate MGD S1 12060 WT	KM507344.1	KM507340.1	KM507338.1	Kenya	2009
Bunyamwera virus strain 46A 122	MH484290.1	MH484289.1	MH484288.1	Kenya	2006
Bunyamwera virus strain 84Brakna ME	MT731757.1	MT731756.1	MT731755.1	Mauritania	2015
Bunyamwera virus strain ATCC(R) VR 87	MZ773507.1	MZ773506.1	MZ773505.1	Uganda	1943
Bunyamwera virus strain GSA S4 11232 WT	KC512400.1	KC512389.1	KC512393.1	Kenya	2009
Calovo virus strain 134	KJ542624.1	KJ542625.1	KJ542626.1	Czech Republic	1963
Calovo virus strain 138 pool 468	KC608157.1	KC608156.1	KC608155.1	Yugoslavia	1983
Calovo virus strain 8020	KJ542630.1	KJ542631.1	KJ542632.1	Slovakia	1975
Calovo virus strain 8040	KJ542633.1	KJ542634.1	KJ542635.1	Slovakia	1975
Calovo virus strain JAn (MS3)	KJ542627.1	KJ542628.1	KJ542629.1	Croatia	1969
Ilesha ILESHA/8e	KC608151.1	KC608150.1	KC608149.1	Senegal	1972
Ilesha KO/2	MT272830.1	MT272831.1	MT272832.1	Nigeria	1967
Ilesha R5964	NC_043585.1	NC_043586.1	NC_043587.1	NA	NA
Kairi virus strain BeAr8226	KR260740.1	KR260739.1	KR260738.1	Brazil	1957
Ngari virus isolate GSA TS7 5170 WT	KM507341.1	KM514677.1	KM507336.1	Kenya	2009
Ngari virus isolate ISL TS2 5242 WT	KM507342.1	KM514678.1	KM507334.1	Kenya	2009
Ngari virus isolate KE B02	ON755203.1	ON755197.1	ON755193.1	Kenya	2020
Ngari virus isolate KE B35	ON755202.1	ON755196.1	ON755192.1	Kenya	2020
Ngari virus isolate KE C166	ON755201.1	ON755199.1	ON755195.1	Kenya	2020
Ngari virus isolate KE O93	ON755200.1	ON755198.1	ON755194.1	Kenya	2020
Ngari virus isolate TND S1 19801 WT	KM507343.1	KM514679.1	KM507335.1	Kenya	2010
Ngari virus strain 15Guidimaka ME	MT747974.1	MT747973.1	MT747972.1	Mauritania	2015
Ngari virus strain 428Trarza ME	MT747977.1	MT747976.1	MT747975.1	Mauritania	2015
Ngari virus strain 9800521	JX857325.1	JX857326.1	JX857327.1	Somalia	1998
Ngari virus strain 9800535	JX857328.1	JX857329.1	JX857330.1	Kenya	1998
Ngari virus strain Adrar	KJ716848.1	KJ716849.1	KJ716850.1	Mauritania	2010
Ngari virus strain Dakar D28542 4e	KC608154.1	KC608153.1	KC608152.1	Senegal	1980
Ngari virus strain SUD HKV141	JX857322.1	JX857323.1	JX857324.1	Sudan	1988
Ngari virus strain SUD HKV66	JX857319.1	JX857320.1	JX857321.1	Sudan	1988
